# The influence of hydrogen on plasticity in pure iron—theory and experiment

**DOI:** 10.1038/s41598-020-66965-z

**Published:** 2020-06-23

**Authors:** Peng Gong, Ivaylo H. Katzarov, John Nutter, Anthony T. Paxton, W. Mark Rainforth

**Affiliations:** 1 Department of Materials Science and Engineering, University of Sheffield, Mappin Street, Sheffield, S1 3JD UK; 2 Department of Physics, King’s College London, Strand, London, WC2R 2LS UK; 3 Bulgarian Academy of Sciences, Institute of Metal Science, 67, Shipchenski prohod Str., 1574 Sofia, Bulgaria

**Keywords:** Mechanical properties, Materials science, Structural materials, Metals and alloys

## Abstract

Tensile stress relaxation is combined with transmission electron microscopy to reveal dramatic changes in dislocation structure and sub structure in pure *α*-Fe as a result of the effects of dissolved hydrogen. We find that hydrogen charged specimens after plastic deformation display a very characteristic pattern of trailing dipoles and prismatic loops which are absent in uncharged pure metal. We explain these observations by use of a new self consistent kinetic Monte Carlo model, which in fact was initially used to *predict* the now observed microstructure. The results of this combined theory and experimental study is to shed light on the fundamental mechanism of hydrogen enhanced localised plasticity.

## Introduction

The subject of hydrogen influence on the mechanical behaviour of steel is hugely controversial. On the other hand the dramatic effects of hydrogen on the mechanical integrity of engineering structures is well documented^1^ and if society is to enter a future hydrogen economy the problem must be tackled head on. The violent reduction in fracture toughness of steel as a consequence of dissolved hydrogen at the level of some atomic parts per million (appm), at the broadest level of current understanding is either the result of a loss of cohesive strength (the HEDE hypothesis) or the consequence of enhanced localised plasticity (the HELP hypothesis). Other theories such as the role of accumulated vacancy damage or the emission of dislocations from crack surfaces have also been proposed^2^. One of the striking features of the problem has been a lack of detailed confirmation of observation with theory and modelling; and vice versa. A particular difficulty arises from the putative elastic shielding of dislocation strain fields due to hydrogen. This is well documented both in elegant electron microscopy observations^3,4^ and sophisticated theoretical treatments in linear elasticity^5,6^. The proposal that HELP is a consequence of elastic shielding by Cottrell atmospheres is untenable in steel because the solubility of hydrogen in body centred cubic *α*-Fe is about six orders of magnitude too small for the effect to be measurable^3,7,8^. Conversely it has been proposed that the hydrogen trapped locally in the cores of dislocations is responsible for the enhanced plasticity^8^. Furthermore it is not obvious that hydrogen will increase dislocation mobility under all circumstances. In fact hydrogen may increase or decrease dislocation velocity depending on the conditions of hydrogen concentration, temperature and applied stress in pure *α*-Fe^9^.

Here, we present for the first time a self consistent kinetic Monte Carlo model that is able to predict average dislocation velocity and to simulate microstructural development that arises from hydrogen self pinning effects. We confirm predictions of the model by transmission electron microscopy (TEM) observations. In both cases we use pure *α*-Fe, and we match as closely as possible the experimental and modelling conditions. Furthermore we make contact between our calculations and recently published measurements of activation volume^10^ and we find a striking accord between experiment and theory. Finally, we conclude with speculations about the role of hydrogen in the generation of dislocation cellular structure which make contact with modern theories of work hardening^11^.

## Theoretical

### Introduction and background

At the heart of the simulation of hydrogen effects on plasticity is the model that is used to describe the connection between background, or nominal, hydrogen concentration, *C*_H_, (here defined in units of atomic parts per million, appm) and either the flow stress or the average dislocation velocity, ${\bar{v}}_{{\rm{dis}}}$. At the simplest level as used in typical discrete dislocation dynamics simulations or crystal plasticity finite element models, simple ad hoc assumptions are used^12,13^. However, ${\bar{v}}_{{\rm{dis}}}$ is a complex function of *C*_H_, and depending on applied stress and temperature ${\bar{v}}_{{\rm{dis}}}$ can be both *enhanced* and *reduced* depending on the background hydrogen concentration^14^. In earlier work^14^, two of us developed an off-lattice kinetic Monte-Carlo method to calculate the velocity of screw dislocations in *α*-Fe based upon first principles calculations of kink-pair formation energies^9^. In this model a number of quite serious approximations are made, namely, (*i*) the kink pair formation energy is affected only by hydrogen ahead of the dislocation in the glide plane; (*ii*) kink velocity is only affected by hydrogen behind the dislocation in the glide plane; (*iii*) hydrogen is assumed to remain fixed in place during kink-pair formation and migration; (*iv*) the time for segments of dislocation to move between Peierls valleys is assumed greater than the hydrogen jump time within the dislocation core. In spite of its simplicity that model was able to predict dislocation velocity as a function of temperature, stress, *τ*, and nominal hydrogen concentration and it was shown that such a function is non monotonic and that the effect of hydrogen can be to increase or decrease dislocation velocity, depending on conditions; and that the change in velocity compared to pure *α*-Fe at 300 K and *τ* = 100 MPa increases by more than a factor of 10 up to 5 appm and then decreases to less than the velocity in pure *α*-Fe at 20 appm. In addition, the simulations^14^ *predicted* that under most conditions of hydrogen -loaded *α*-Fe a moving screw dislocation will leave a trail of debris made up of rows of prismatic loops. The central result of the present paper is that *we have found these loops* in electron microscope images of deformed, hydrogen-charged *α*-Fe. On the other hand the model was not able to reproduce activation volume measurements^10^ which indicate a minimum in the dislocation velocity as a function of hydrogen concentration at about 10 appm at 300 K. We present here a new model which we call “self consistent kinetic Monte-Carlo” (SCkMC) which permits a dynamic non equilibrium distribution of hydrogen about the moving dislocation core. Specific new features of the model are, (*i*) simultaneous kink nucleation, migration and hydrogen jumping; (*ii*) kink pair formation energy affected by all hydrogen within the core; (*iii*) a non equilibrium distribution of hydrogen which depends on temperature and average dislocation velocity; (*iv*) kink pair formation energy depends on average dislocation velocity; (*v*) mobile hydrogen during glide—although the *total* hydrogen occupancy within the core is assumed fixed.

### Line tension model

The SCkMC is predicated on a parameterised line tension model^9,15^. We imagine a long dislocation lying in its Peierls valley, a segment of which has migrated towards or into the next Peierls valley so as to make an incipient or complete kink pair. The dislocation is divided into bins of width *b*, the Burgers vector, along its length and a variable *x*_*j*_ is assigned to describe the deviation of the segment lying in the *j*^th^ bin from the dislocation’s original position in the Peierls valley—the elastic center of the dislocation. There is a periodic Peierls energy landscape described by an energy function, *E*_*p*_(*x*_*j*_). The energy per unit length of dislocation is then prescribed in the following line tension expression^9^,

1 $$\begin{array}{rcl}E & = & \sum _{j}\,{E}_{j}\\  & = & \frac{1}{2}K\sum _{j}\,{({x}_{j}-{x}_{j+1})}^{2}+\sum _{j}\,{E}_{p}({x}_{j})+\sum _{j}\,{\varepsilon }_{1pq}{\tau }_{pr}{b}_{r}{\xi }_{p}{x}_{j}-\sum _{jk}\,{E}_{{\rm{H}}}(|{x}_{j}-{x}_{k}^{{\rm{H}}}|)\end{array}$$

The first term describes the energy penalty for two bins which have different amounts of deviation from the original Peierls valley towards the next and *K* is the associated “spring constant”. The second term is the energy of the segment *j* depending on its height in the Peierls landscape. The third term, with an implicit sum from 1 to 3 over {*pqr*}, is the 1-component (perpendicular to [111]) of the Peach–Kohler force arising from a local stress *τ*_*pq*_ times the displacement of the *j*^th^ segment of dislocation having a line sense **ξ**. This term “tilts” the corrugated energy landscape so that the Peierls valley ahead of the dislocation is lower in energy that the one behind, and provides the driving force for glide. The final term expresses the energy associated with a hydrogen atom that is trapped at a position at a distance $|{x}_{j}-{x}_{k}^{{\rm{H}}}|$ from the core, in which ${x}_{k}^{{\rm{H}}}$ is the position of the *k*^th^ hydrogen atom relative to the elastic centre.

### Dynamics of the long straight dislocation

We first examine the motion of a long straight dislocation, its line moving as a whole. And in the next section we address the actual situation of glide by the Peierls mechanism of kink pair creation and kink migration^16^. Density functional theory (DFT) calculations have identified two core structures of the $\frac{1}{2}[111]$ screw dislocation, the so called “easy core” (EC), which is the stable, low energy configuration, and the “hard core” (HC) which is metastable^15,17^. The HC is very close in configuration to the “saddle point” (SP) core^15,17^. DFT calculations furthermore show that hydrogen binds strongly to the EC with three equivalent sites having binding energies of *E*_*i*_ = 256 meV in the so called *E*_1_/*E*_2_ basin, three in the *E*_3_/*E*_4_ basin having *E*_*i*_ = 201 meV and six in the *E*_7_/*E*_8_ basin with *E*_*i*_ = 77 meV^9^. The strongest binding sites for the HC are one in the *H*_0_/*H*_1_ basin located at the centre of the core with *E*_*i*_ = 390 meV, and six binding sites denoted *H*_2_ having *E*_*i*_ = 189 meV^9^. As a dislocation moves from EC to HC to EC the *E*_1_/*E*_2_ traps sites ahead of the dislocation line transform into *H*_0_/*H*_1_ sites and finally the hydrogen occupies *E*_1_/*E*_2_ traps sites behind the dislocation line.

When the dislocation is lying in its equilibrium Peierls valley the probability of occupancy, *χ*_*i*_, of a trap site, *i*, is determined by the McLean isotherm^18^,

2 $${\chi }_{i}=\frac{\frac{1}{6}{C}_{0}\,{e}^{{E}_{i}/kT}}{1+\frac{1}{6}{C}_{0}\,{e}^{{E}_{i}/kT}}$$

in which *C*_0_ = 10^−6^ *C*_H_ is the nominal number of hydrogen atoms per Fe atom, and the factor 1/6 accounts for there being six tetrahedral sites per bulk Fe atom. Here, *k* is the Boltzmann constant and *T* is the absolute temperature. If we take a sum over all the trap sites in the dislocation core, we will define

3 $${\chi }_{t}=\sum _{i}\,{\chi }_{i}={\rm{constant}}$$

as the total hydrogen occupancy of the core sites; and we will assume throughout that *this is constant*, that is, hydrogen will redistribute dynamically between trap sites during glide but overall the dislocation will not absorb or reject hydrogen; we also only allow hydrogen to redistribute among traps within a plane perpendicular to the dislocation line, in view of the very slow hydrogen pipe diffusivity^19^. In the case of slow glide, and the maintenance of equilibrium, then as a long straight dislocation moves between two Peierls valleys, we may define the occupation probability, ${\chi }_{i}^{{\rm{e}}}$, of trap site *i* as^20^,

4 $${\chi }_{i}^{{\rm{e}}}(x)=\frac{{\chi }_{t}{e}^{-{E}_{i}(x)/kT}}{\sum _{j}\,{e}^{-{E}_{i}(x)/kT}}$$

Here 0 < *x* < *h*, if $h=a\sqrt{2/3}=2.34$ Å is the period of the Peierls potential on the $(\bar{1}10)$ plane, so that *x* describes the position of the dislocation line with respect an origin at the EC elastic centre. As the dislocation glides hydrogen will redistribute between trap sites, which themselves distort and therefore whose trap depth, *E*_*i*_(*x*), varies with *x*. We parameterise *E*_*i*_(*x*) by fitting and interpolation of DFT data^9^. Once that is done, then in association with the line tension model (1) we have a complete description of the energetics of the dislocation as a function of *x* and the total occupancy, *χ*_*t*_, *for the moment only* in two limiting cases: (**a**) equilibrium, slow glide in which traps are occupied according to (4), and (**b**) fast glide, in which all hydrogen atoms are fixed in the traps they occupy in the EC initial state before glide.

**Figure 1:**
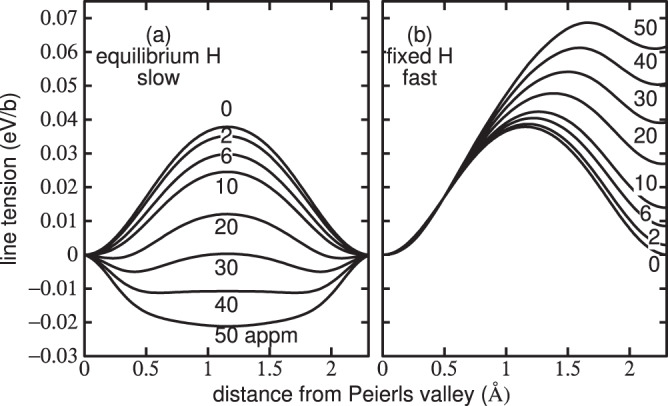
Peierls potential: the potential energy in units of eV per Burgers vector of a long straight $\frac{1}{2}[111]$ screw dislocation as a function of distance between one Peierls valley and the next. (**a**) Limiting case of slow motion: the hydrogen remains in equilibrium and moves reversibly between *E*_1_/*E*_2_ basins. At the saddle point the hydrogen is trapped at the *H*_0_/*H*_1_ basin near the saddle point. Note, how as hydrogen concentration is increased above 30 appm the saddle point core structure becomes more stable than the easy core. (**b**) Limiting case of high dislocation velocity: the hydrogen remains behind in a trap site of high energy compared to the *E*_1_/*E*_2_ basin hence the line tension is greater after glide by one repeat distance than before. The curves are labelled with the nominal background hydrogen concentration, *C*_H_. Temperature is 300 K.
- In the limit of *rapid glide* the hydrogen atoms are kept fixed during the movement of a dislocation between Peierls valleys; then as the dislocation moves, hydrogen that was trapped in deep traps may not jump into the newly created traps, but instead remains behind in sites of higher potential energy; hence the Peierls barrier increases continually with *C*_H_ and the initial and final positions of the dislocation line have not the same energy: the profile is asymmetric as shown in Fig. 1(b).

- Figure 1(a) shows potential energy profiles in the equilibrium limit of a slowly moving dislocation. At *C*_H_ = 0 the profile is typical of a calculated Peierls barrier^21^. The Peierls barrier shown predicted by our model is consistent with the measured estimate of 37 meV/b^16^. The barrier becomes smaller as *C*_H_ is increased because hydrogen is stabilising the saddle point core as the *E*_1_/*E*_2_ traps distort into *H*_0_/*H*_1_ traps. In fact the effect is strong enough so that when *C*_H_ exceeds 30 appm the saddle point core is lower in energy than the easy core and their roles are reversed; this is because the total energy gained by hydrogen in deeper traps overwhelms the penalty in core energy. In this way the Peierls barrier is reduced to close to zero and then increases again. However above about 30 appm hydrogen the saddle point is at the EC, and the minimum is in the HC configuration.

A highly relevant conclusion is that trapped hydrogen serves to *stabilise the hard core with respect to the easy core*, so that hydrogen is able to trigger a core transformation which strongly modifies the Peierls barrier.

**Figure 2:**
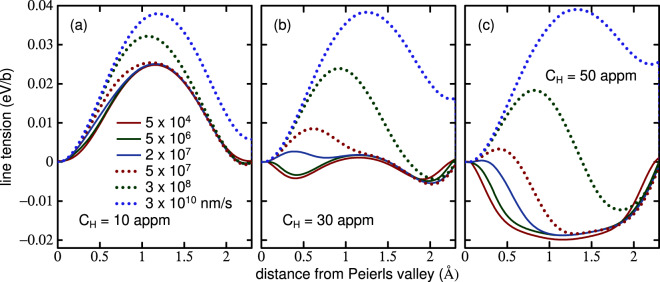
Potential energy of a long straight $\frac{1}{2}[111]$ screw dislocation as in Fig. 1. Panels (a), (b) and (c) show solutions using the continuity Eq. (5) at nominal hydrogen concentrations of 10, 30 and 50 appm respectively. In cases where the saddle point is of lower energy than the EC end points, the Peierls barrier is inferred by taking the end points as saddle point energies and the stable core to be the HC. Curves for dislocation velocities, *v*, between 5 × 10^4^ and 3 × 10^10^ nm s^−1^ are shown. Temperature is 300 K.

The results in Fig. 1 suggest to us that the actual profile will be somewhere in between the two limits, the departure from equilibrium being controlled by the uniform dislocation velocity, *v*. Therefore we seek a theory that will predict the profile as a function of *v*. Because of the finite speed of the dislocation, we expect that the probability of occupancy of trap *i*, *χ*_*i*_(*x*), differs from its equilibrium value (4), ${\chi }_{i}^{{\rm{e}}}(x)$. For any of the ten strongest binding sites we find the following continuity equation,

5 $$\frac{\partial {\chi }_{i}(x,v)}{\partial t}=v\frac{\partial {\chi }_{i}(x,v)}{\partial x}=({\chi }_{i}^{{\rm{e}}}(x)-{\chi }_{i}(x,v))f{e}^{-{E}_{i}(r)/kT}$$

where *f* is an “attempt frequency” for hydrogen to escape from the *i*^th^ trap^22^. By solving (5), subject to the condition (3) that the total hydrogen occupancy remains constant, we may determine the potential energy of the dislocation as a function of position between two Peierls valleys at velocity, *v*. We show these data in Fig. 2.

We observe that at the critical *C*_H_ of 30 appm where the Peierls barrier for low velocity is close to zero, the actual barrier is strongly dependent on the velocity and only vanishes in the slow, equilibrium limit.

### Dynamics by kink pair creation and migration

The $\frac{1}{2}[111]$ screw dislocation in bcc transition metals is characterised by its non planar, non degenerate core structure^23^ which means that even at the lowest temperatures, its glide is via a *Peierls mechanism*, namely the process of kink pair creation followed by kink migration^20^. Kink pair generation is thermally activated. We therefore turn now to the actual problem of predicting ${\bar{v}}_{{\rm{dis}}}$ within the Peierls mechanism^16^. Note that ${\bar{v}}_{{\rm{dis}}}$ is the dislocation velocity averaged over the multitude of the kink-pair creation and separation processes; while *v* in the last section is the notional velocity of a long straight dislocation moving uniformly between Peierls valleys.

#### Kink pair creation

The screw dislocation does not lie quiescent in its Peierls valley; fluctuations produce random events in which a small section deviates towards a neighbouring Peierls valley. Mostly this produces an “incipient” kink pair which annihilates due to elastic attraction of the kinks. A stable kink pair is one that has sufficient distance between the kinks, which we take to be about 30*b*^14,15^, that elastic attraction is small enough to allow the kink pair to survive and its halves to separate under the local stresses they encounter. The formation of a stable kink pair is a result of numerous acts of kink-pair nucleation, annihilation, and increasing distance between kinks under the action of the applied shear stress. We do not consider all these processes explicitly in our simulations. The rare event of formation of a stable kink pair, which separates under the resolved shear stress is treated using the kinetic Monte-Carlo procedure described elsewhere^14,24,25^.

The reason for requiring a *self consistent* theory is that trapped hydrogen will strongly modify the kink pair formation enthalpy, *E*_kp_, and that the location of hydrogen in traps will depend on how fast the dislocation is moving. Hence *E*_kp_ is a function of ${\bar{v}}_{{\rm{dis}}}$ since it depends on the rate at which hydrogen is distributed among trap sites as the dislocation glides. For a given resolved shear stress, *τ*, and an assumed average velocity, ${\bar{v}}_{{\rm{dis}}}$, using the line tension model and data such as in Fig. 2 the energy, ${E}_{j}({C}_{{\rm{H}}},x,{\bar{v}}_{{\rm{dis}}})$, of a dislocation segment, Eq. (1), of length *b* and at a distance *x* from the EC elastic centre in the initial Peierls valley, can be calculated. Then using linear, non-singular elastic theory^20,25^ and the “nudged elastic band” (NEB) method^26^, we may calculate the kink pair formation enthalpy, ${E}_{{\rm{kp}}}({C}_{{\rm{H}}},\tau ,{\bar{v}}_{{\rm{dis}}})$. *However E*_kp_ is a function of ${\bar{v}}_{{\rm{dis}}}$ while ${\bar{v}}_{{\rm{dis}}}$ is a function of *E*_kp_: ${E}_{{\rm{kp}}}={E}_{{\rm{kp}}}({\bar{v}}_{{\rm{dis}}})$ and ${\bar{v}}_{{\rm{dis}}}={\bar{v}}_{{\rm{dis}}}({E}_{{\rm{kp}}})$. To make progress and to find a self consistent solution, we assume that the average speed is constant, and

6 $${\bar{v}}_{{\rm{dis}}}({E}_{{\rm{kp}}})=\frac{h}{{t}_{r}}$$

(*h* is defined after Eq. (4)) allowing us to define an *average relaxation time* for kink pair formation,

7 $${t}_{r}={f}_{{\rm{kp}}}^{-1}{e}^{{E}_{{\rm{kp}}}({C}_{{\rm{H}}},\tau ,v)/kT}$$

where *f*_kp_ is an attempt frequency. We treat this as a disposable parameter which we adjust to obtain the measured dislocation velocity in pure *α*-Fe^16^. We find *f*_kp_ = 2.31 × 10^9^ s^−1^. In order to solve (6) and (7), and to determine *E*_kp_ at given *C*_H_ and *τ*, we proceed with the following iterative process.

1. Assume an initial *E*_kp_.
2. Calculate the corresponding ${\bar{v}}_{{\rm{dis}}}$ using (6) and (7).
3. Determine the distribution of hydrogen from the continuity Eq. (5), subject to (3); and calculate the segment energy, $E({C}_{{\rm{H}}},x,{\bar{v}}_{{\rm{dis}}})$ from the line tension model.
4. Calculate *E*_kp_ using the NEB and go to step 2.

**Figure 3:**
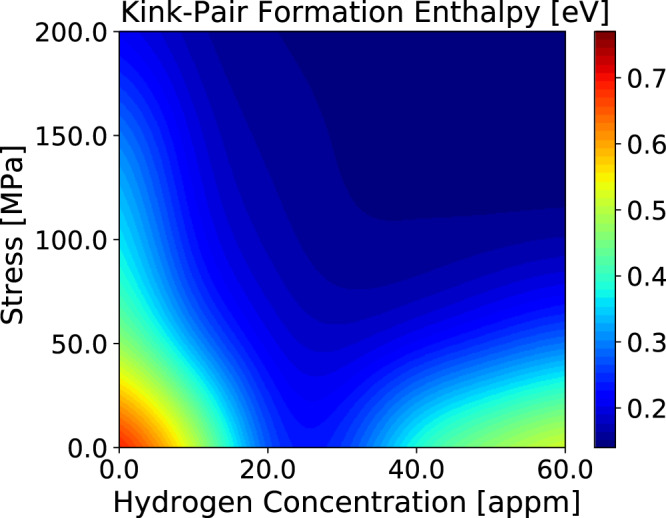
Kink pair formation enthalpy, *E*_kp_, as a function of resolved shear stress, *τ* and hydrogen concentration; calculated by iterative solution of Eqs. (6) and (7).

This process is iterated until *E*_kp_ calculated in step 4 is no longer changing to within some tolerance. Figure 3 shows the results of the iterative procedure.

We may interpret Fig. 3 in the following way. At high stress, *E*_kp_ is uniformly small because the applied stress acts to drive a dislocation into the next Peierls valley and this dominates the process of glide. At low stress we observe a large *E*_kp_ at low *C*_H_, the largest being that of pure *α*-Fe and zero stress. As *C*_H_ increases, *E*_kp_ decreases, consistent with the calculations shown in Fig. 1. *E*_kp_ reaches a minimum at *C*_H_ ≈ 30 appm in Fig. 3 as predicted in Fig. 1 and this minimum in *E*_kp_ is a consequence of the hydrogen-induced core transformation from EC to HC. As *C*_H_ increases further *E*_kp_ rises as a consequence of the increasing Peierls barrier—but now the barrier is at the easy core configuration and the Peierls valley corresponds to the HC.

#### Kink migration

Glide is a two step process. After the formation of a stable double kink the two kinks will separate in opposite directions. In pure metal, the kink migration or *secondary Peierls barrier* is low and is not thermally activated. However hydrogen and other interstitials change that. If a hydrogen atom is trapped in the *E*_1_/*E*_2_ basin just behind the dislocation line and a kink sweeps past, then that hydrogen ends up in a higher enthalpy trap site^9^, which implies that thermal activation is then required for the kink to proceed. We do not need to rehearse the kMC procedure here since we use the identical scheme as described earlier^14^. However we should underline the *physics* here since it is essential in appreciating the present findings. In the case of pure *α*-Fe, a screw dislocation of typical length of about 1000*b* will glide as a unit as in face centred cubic metals (albeit by thermal activation of kink pairs) since the kink migration speed is so fast that a kink pair has separated to the ends of the dislocation before the next kink pair is activated^14^. Hence kink collision does not occur. The situation is very different if the kinks suffer solute drag due to hydrogen and other interstitials. A key fact is that kink pairs are created on any one of the three $\{\bar{1}10\}$ glide planes in the zone of the [111] Burgers vector. *If two kinks on different glide planes collide* the resulting defect is an edge jog which is sessile. Our findings earlier^14^, which we confirm here, are that such jogs amount to self pinning points which drag out edge dipoles and these dipoles will pinch out to create a train of prismatic loop debris, *entirely as a consequence of dissolved hydrogen*.

### Results of the self consistent kinetic Monte-Carlo simulations

#### Average dislocation velocity

**Figure 4:**
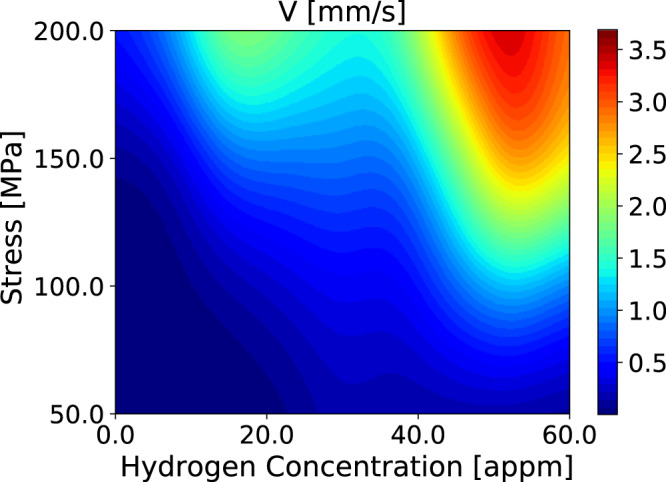
Average dislocation velocity, ${\bar{v}}_{{\rm{dis}}}$, calculated within the self consistent kinetic Monte-Carlo model using *E*_kp_ from Fig. 3.

Conditions of the present self consistent kMC simulations are identical to those of the earlier non self consistent modelling^14^. Temperature is 300 K. Rather than using a kink pair formation energy that depends only on stress, *C*_H_ and temperature; we now employ *E*_kp_ as a function of ${\bar{v}}_{{\rm{dis}}}$ also as taken from Fig. 3. Average dislocation velocity as a function of stress and *C*_H_ is shown in Fig. 4.

For *τ* < 50 MPa (not shown in Fig. 4), ${\bar{v}}_{{\rm{dis}}}$ increases with *C*_H_, reaching a maximum at *C*_H_ ≈ 25 appm; thereafter ${\bar{v}}_{{\rm{dis}}}$ decreases as a result of the increase in *E*_kp_ (Fig. 3). At *τ* > 100 MPa, ${\bar{v}}_{{\rm{dis}}}$ does not go through a minimum, but increases steadily with *C*_H_ until *C*_H_ ≈ 40 appm at which a rather dramatic increase is found, followed by a decline at higher hydrogen concentrations. The greatest average dislocation velocity, for all stresses, occurs at a nominal hydrogen concentration of about 50 appm. This complex behaviour can be traced in part to the concentration dependence of the kink pair formation enthalpy and the hydrogen -induced core transition from easy core to hard core. If *E*_kp_ is small or vanishing then kink pair formation is easy on all three glide planes in the zone of the Burgers vector, and this leads to increased likelihood of kink pair collisions on different glide planes. Once an immobile jog is created further kinks pile into it, resulting in the formation of superjogs and trailing dislocation dipoles (see Fig. 11). The two arms of the dipole may intersect and recombine by kink pair recombination. Thereby the dipole is unzipped and a straight dislocation in screw orientation is restored. This involved set of operations serves greatly to attenuate the average dislocation velocity as the overall line waits for these events to complete and the dislocation to unpin itself.

#### The development of debris

**Figure 5:**
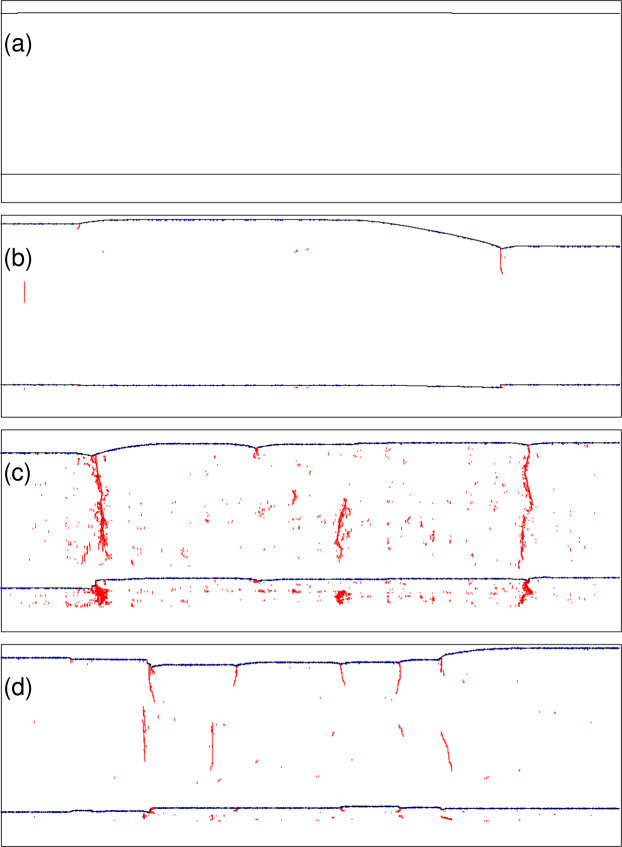
Snapshots of a moving $\frac{1}{2}[111]$ screw dislocation (black line) projected onto $(\bar{1}10)$ (upper line) and $(11\bar{2})$ (lower line) planes. The red lines indicate trailing debris. Blue dots represent the positions of hydrogen atoms. *τ* = 100 MPa, *T* = 300 K (**a**) *C*_H_ = 0, (**b**) *C*_H_ = 10 appm, (**c**) *C*_H_ = 30 appm, (**d**) *C*_H_ = 50 appm.

These observations are illustrated in Fig. 5 which show simulations at a resolved shear stress, *τ* = 100 MPa, and *T* = 300 K. Very similar microstructures are observed in a range of stresses from 10 MPa to 200 MPa. In each panel the upper black line shows a snapshot of a moving $\frac{1}{2}[111]$ screw dislocation projected onto the primary $(\bar{1}10)$ glide plane, while the lower black line shows the *same* dislocation at the same time projected onto the perpendicular $(11\bar{2})$ plane. This second projection serves to indicate the extent to which the dislocation deviates from its primary glide plane into the two cross slip planes in the [111] zone.

- At *C*_H_ = 0, panel (a) illustrates the point made earlier that kink velocity is high and the dislocation moves as a straight line (although at *T* = 400 K kink pair generation is sufficiently frequent that kink collisions do occur and some debris is observed^14^).
- At *C*_H_ = 10 appm, Fig. 5(b), *E*_kp_ is large (Fig. 3) and nucleation on cross slip planes is rare so that kink collisions on different slip planes is less likely—some debris is seen and the dislocation is not straight in its primary slip plane, however deviation onto a cross slip plane is limited.
- At *C*_H_ = 30 appm *E*_kp_ is small (Fig. 3) and nucleation on cross slip planes is commonplace: there is much debris observed and significant cross slip of the dislocation onto secondary glide planes.
- As *C*_H_ is further raised to 50 appm, Fig. 5(d), *E*_kp_ is raised again (Fig. 3), the equilibrium core structure is the hard core and kink pair generation on the cross slip planes is again less common—less debris accumulates than at *C*_H_ = 30 appm.

#### The “30 appm anomaly” and comparison with experiment

**Figure 6:**
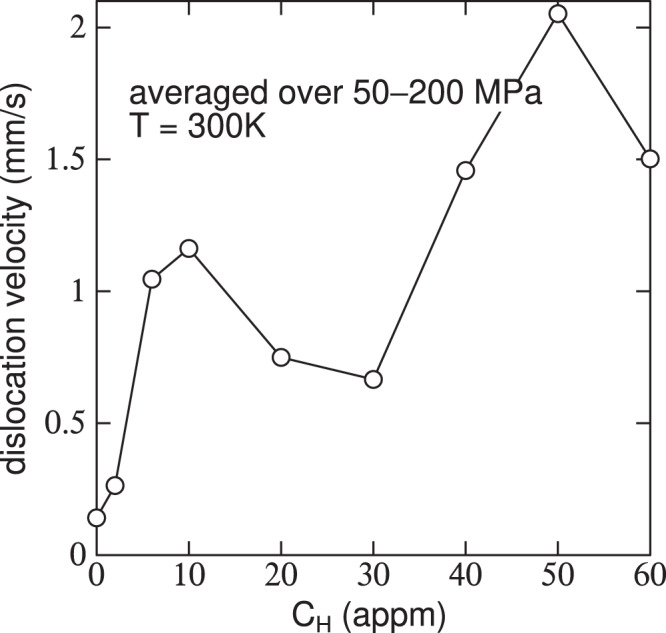
Calculated dislocation velocity, ${\bar{v}}_{{\rm{dis}}}$, averaged over resolved shear stresses in the interval 50–200 MPa.

It is very clear from all the results presented above that there is a strong non monotonic dependence of ${\bar{v}}_{{\rm{dis}}}$ on *C*_H_ with an “anomaly” occurring around *C*_H_ = 30 appm. The reason for this is the reduction in kink pair formation enthalpy and the associated core transformation from EC to HC. This effect is revealed most simply in Fig. 6 which is a plot of ${\bar{v}}_{{\rm{dis}}}$ averaged over resolved shear stresses in the interval 50–200 MPa. In an experiment at a nominal shear stress of, say, 50 MPa (as in our measurements to be described below) the actual stress experienced by a dislocation varies rather widely in a range about the nominal stress, on account of microstructural features including grain boundaries, pile ups, load shedding across grains. Therefore it makes sense to take an average as we do here. The evident dip in ${\bar{v}}_{{\rm{dis}}}$ is mirrored in measurements of the components of the activation volume for tensile deformation of hydrogen charged *α*-Fe. The method used is stress relaxation^27^. The applied shear stress is divided into a thermally activated contribution, *τ*_eff_, and a term, *τ*_*μ*_, that depends on temperature only through the *T*-dependence of the shear modulus^28^,

$${\tau }_{{\rm{app}}}={\tau }_{\mu }+{\tau }_{{\rm{eff}}}$$

The strain rate as a function of temperature is given in terms of an activation free energy, *G*, of the strain rate, $\dot{\gamma }$, defined through,

$$\dot{\gamma }={\dot{\gamma }}_{0}\,{e}^{-G/kT}$$

where ${\dot{\gamma }}_{0}$ is given by the Orowan equation^27^ and depends on the average dislocation velocity. The “effective” activation volume is

$${V}_{{\rm{eff}}}=-\frac{dG}{d{\tau }_{{\rm{eff}}}}$$

**Figure 7:**
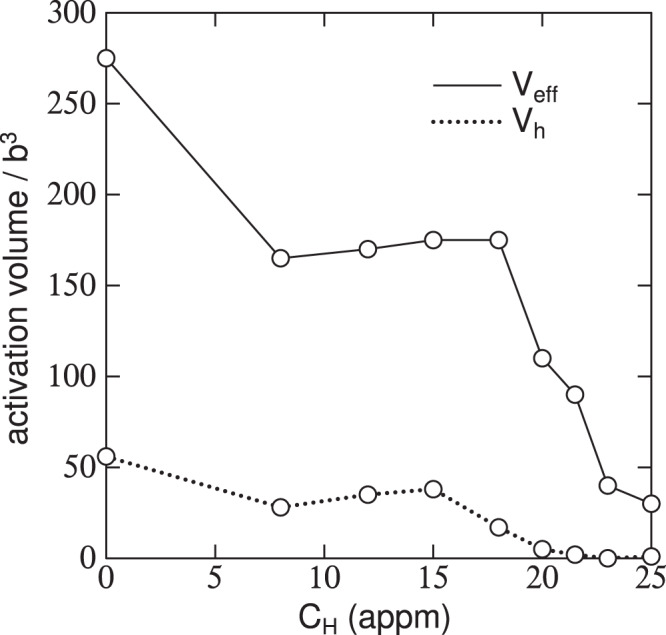
Measured components of the activation volume for plastic shear in hydrogen loaded pure *α*-Fe. After Wang *et al*.^10^.

What is measured is the consequence of the total applied shear stress, namely an “apparent” activation volume,

8 $${V}_{{\rm{app}}}={V}_{{\rm{eff}}}\left(1+S{\prime} \frac{d{\tau }_{\mu }}{d\gamma }\right)={V}_{{\rm{eff}}}+{V}_{{\rm{h}}}$$

where *S*′ is the compliance of the specimen plus loading train in the tensometer. Stress relaxation tests allow the two terms, the effective and the “hardening” activation volumes to be identified separately. Figure 7 shows such measurements, taken from Wang *et al*.^10^. For reasons we give in the Discussion Section, namely the difficulty in assessing hydrogen concentration, we associate the minimum in Fig. 6 at about 30 appm with the minimum in Fig. 7 at about 10 appm.

It is notable that the anomaly is associated with a *decrease* in the Peierls barrier and a *decrease* in both the average dislocation velocity *and* the effective activation volume (or, at least, a plateau in *V*_eff_). We resolve this apparent contradiction as follows. Since at this hydrogen concentration the Peierls barrier and kink pair formation enthalpy is close to zero, one would *expect* that ${\bar{v}}_{{\rm{dis}}}$ would be fast. However because of the peculiar three fold core structure of screw dislocations in *α*-Fe, the fact that *E*_kp_ is nearly vanishing implies that kink pair generation is very rapid on both the primary glide plane *and* the two cross slip planes. This vastly increases the likelihood of kink collisions on dissimilar slip planes leading to frequent creation of jogs and superjogs, the generation of debris and the subsequent reduction in the *average* dislocation velocity. Because the thermal activation barrier is small this also reflects on the activation volume which consequently also reached a minium or plateau as seen in Fig. 7.

## Experimental

### Materials and Methods

99.99% pure iron was purchased from Goodfellow, Cambridge. Samples 100 × 100 mm by 2 mm thick were received in the cold rolled condition. Samples were annealed at 650 °C for one hour in order to fully recrystallise the structure and ensure as low a starting dislocation density as possible. Specimens for stress relaxation tests were manufactured with a sample length of 56 mm, a width in the gauge area of 3 mm, a gauge length of 12.5 mm and a thickness of 2 mm. The starting structure was polycrystalline with an average grain size of 62 *μ*m. Hydrogen charging was undertaken using standard electrochemical techniques. The stress relaxation test specimens were charged using 1 g/L in an aqueous solution of 3 wt% NaCl and 0.3 wt% NH_4_ SCN with a current density of 10 mA cm^−2^ for 48 hours at room temperature. Using thermal desorption spectroscopy we determine the hydrogen concentration to be 30 ± 3 appm. Following charging, samples were immediately subject to repeat stress relaxation tests. Tests were undertaken using a Zwick (BTC T1-FR020 TN A50) universal testing machine. Testing was conducted under displacement control, with a strain rate of 10^−5^ s^−1^. A displacement of 0.3 mm was imposed, which gave a stress of 98 MPa in the sample, which was just beyond the yield point of both charged and uncharged specimens. At this point, the strain was held constant for 30 s allowing stress relaxation. Subsequently the specimen was loaded to the same stress of 98 MPa, and then the strain was held constant again for 30 s. The same cycles were repeated until no relaxation was recorded in the relaxation stage. Stress relaxation data was analysed to determine the values of *V*_eff_ and *V*_h_ (8), for the charged and uncharged specimens. We found *V*_eff_ = 133 ± 13*b*^3^ and *V*_h_ = 11 ± 4*b*^3^ and *V*_eff_ = 127 ± 13*b*^3^ and *V*_h_ = 13 ± 13*b*^3^ in uncharged and charged pure *α*-Fe respectively.

In order to observe dislocation microstructures TEM thin foils were extracted from the stress relaxation specimens. Samples were removed from the centre of the gauge section using standard metallographic techniques. TEM thin foils were prepared by electropolishing using an electrolyte solution of 5% perchloric acid, 35% butoxyethanol and 60% methanol. Scanning Transmission Electron Microscopy (STEM) observations of the thin foil samples were then conducted in the JEOL F200 TEM operated at an accelerating voltage of 200 kV. Orientation mapping was undertaken in the TEM using a Nanomegas ASTAR system, an automated crystal orientation and phase mapping tool using precession electron diffraction. For mapping, the STEM was in spot size 7, with a 10 micron condenser aperture, a precession angle of 0.7 degrees and a step size of 2.5–10 nm depending on the scan area. Diffraction patterns were recorded using the external camera, with an exposure time of 40 ms.

### Experimental results

Scanning transmission electron microscopy (STEM) was used to characterise the dislocation structures before and after charging in the unstrained state, and then after stress relaxation tests, again for charged and uncharged specimens. Care was taken to ensure that the observations of dislocation structures from charged and uncharged specimens were, as close as possible, from the same conditions. Samples were assessed using three techniques, namely, high resolution EBSD, precession electron diffraction to determine orientations and TEM. EBSD mapping was used to select grains which had as close as experimentally possible a similar crystal orientation with respect to the stress axis. Imaging of the dislocation structures was undertaken using STEM close to the [111] zone axis. STEM was preferred over TEM for imaging dislocations as it is better able to resolve individual dislocation lines in dense dislocation walls. Specifically, STEM studies can be performed on thicker specimens than in TEM and image contrast from bend contours and thickness fringes are less pronounced due to the convergent probe.

**Figure 8:**
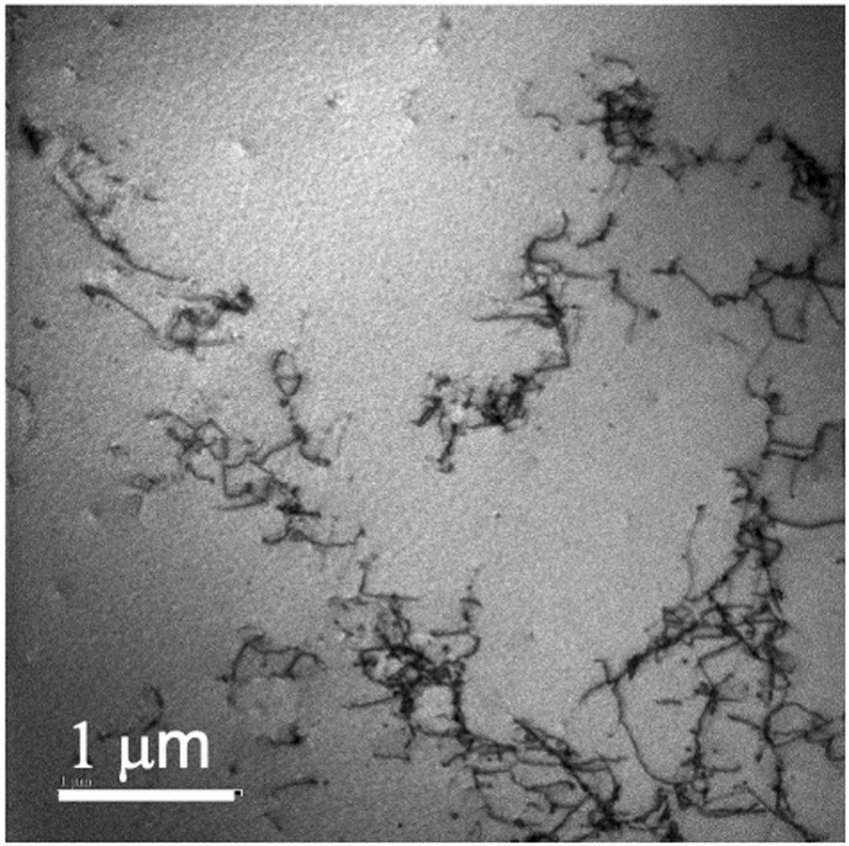
Bright field STEM image showing the hydrogen charged, strain free state.

The dislocation structures in the hydrogen free, strain free samples are as expected, with a low dislocation density, comprising largely homogeneous dislocation distributions. On charging, but without strain, the dislocation density measurably increases, although the total dislocation density remains low. The dislocations tended to be in tangles but individual dislocation lines could still be imaged, Fig. 8, with the results entirely consistent with those of Wang *et al*.^10^.

**Figure 9:**
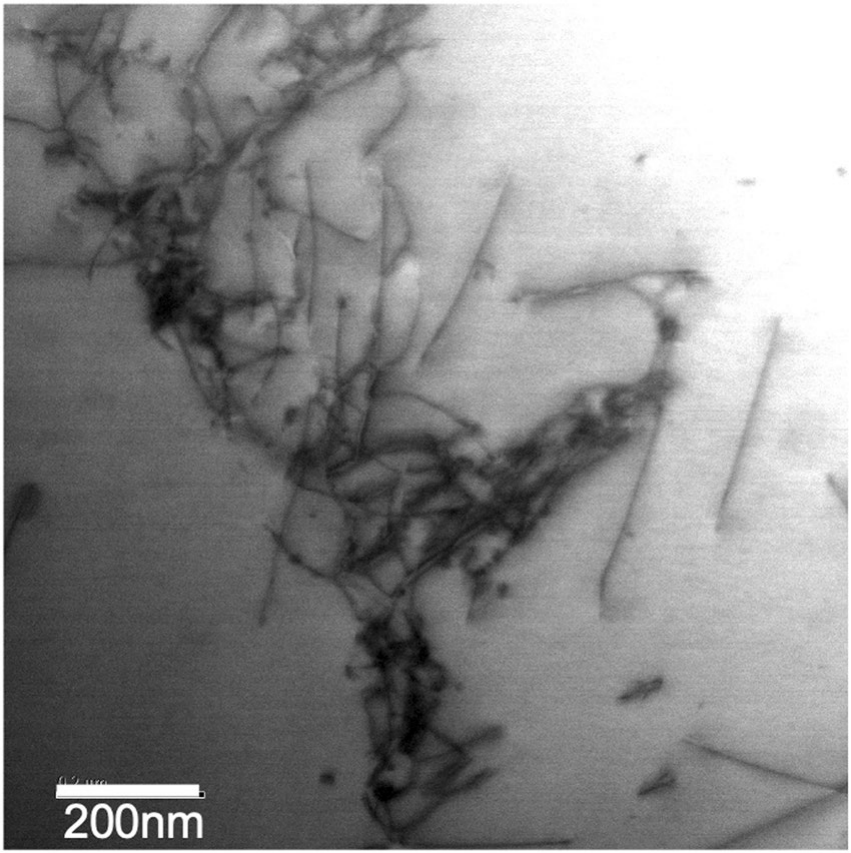
Bright field STEM image from a hydrogen free specimen after tensile stress relaxation testing. Loose dislocation tangles are seen.

**Figure 10:**
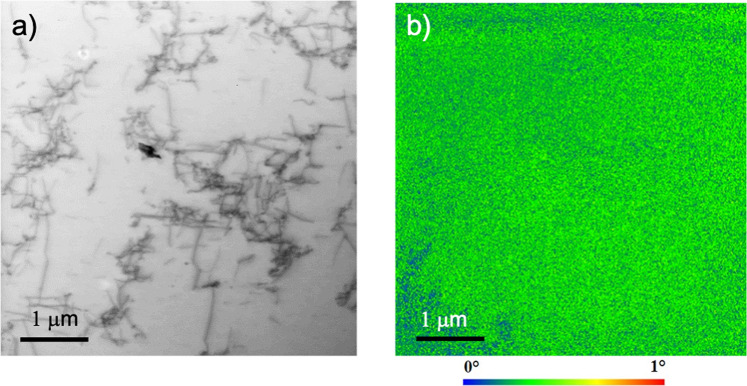
(**a**) Bright field STEM image of an uncharged specimen after tensile stress relaxation showing the dislocation structure. (**b**) Kernel average misorientation image from the same area using precession electron diffraction, showing the absence of lattice curvature.

The change in dislocation structure following stress relaxation testing of the hydrogen free pure iron samples is shown in Fig. 9. Dislocations are arranged as dislocation tangles, but with individual dislocation lines easily imaged in places. No lattice curvature could be measured across these dislocation walls, as shown by the orientation image obtained by precession electron diffraction in Fig. 10.

**Figure 11:**
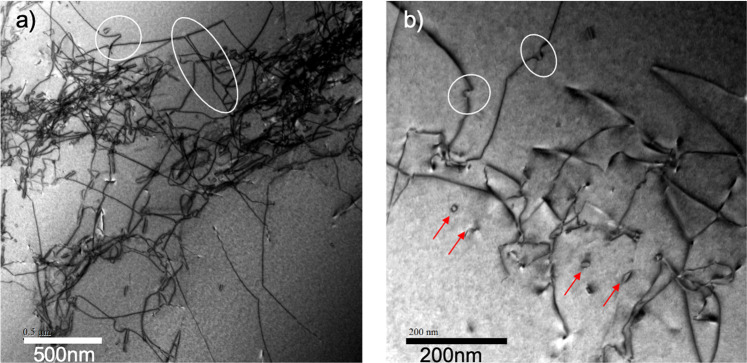
Bright field STEM images of a specimen, hydrogen charged to 30 appm, after tensile stress relaxation. (**a**) At the top centre of the image can be seen a long trailing dipole (circled). If this is in edge orientation then it may find it difficult to unzip. Another instance of self pinning into a V-shape is evidenced at the top left. The dislocations are rather clearly jogged in both images. (**b**) Multiple examples of loop debris are clearly observed (arrowed).

**Figure 12:**
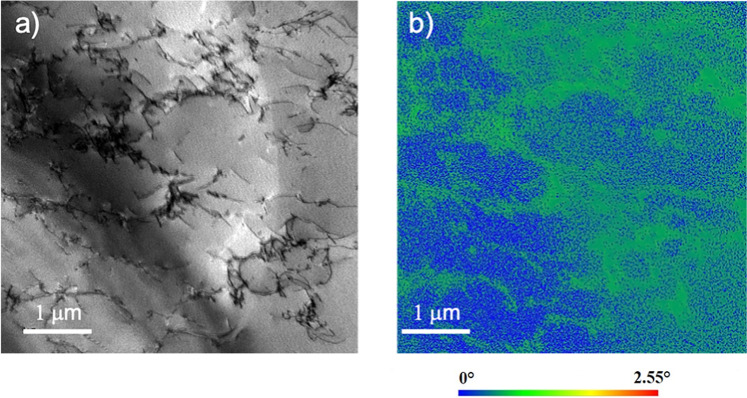
(**a**) Bright field STEM image of the specimen hydrogen charged to 30 appm after tensile relaxation showing the dislocation structure. (**b**) Kernel average misorientation image from the same area as (**a**) using precession electron diffraction, showing the lattice curvature associated with the formation of a rudimentary cell structure.

The dislocation structures for the hydrogen charged stress relaxation samples are markedly different. Dense dislocation tangles are present, Fig. 11. A rudimentary cell structure formed in places, which produced a measurable misorientation across the dislocation walls, as shown by the orientation imaging in Fig. 12. There are numerous examples of jogs and dislocation debris such as prismatic loops. This result is consistent with Fig. 5. As noted before, the dislocation jogs act as self-pinning points, which result in edge dipoles being dragged out, leading to a train of prismatic loops. This effect is purely a result of the dissolved hydrogen in the sample.

## Discussion

### Interpretation of activation volume and the “30 appm anomaly”

Our calculations of average dislocation velocity as a function of hydrogen concentration, Fig. 6, show a deep minimum at about 30 appm where the self pinning of the dislocation attenuates the otherwise increasing ${\bar{v}}_{{\rm{dis}}}$ with *C*_H_ and almost returns ${\bar{v}}_{{\rm{dis}}}$ to that of pure *α*-Fe. This is reflected in the measured effective activation volume from Wang *et al*.^10^ and plotted in Fig. 7. In this plot, *V*_eff_ is seen to rise at about 18 appm, but not quite to reach that of pure *α*-Fe before falling to a small value associated with enhanced ${\bar{v}}_{{\rm{dis}}}$ due to hydrogen. Our own measurements of effective activation volume show that at 30 appm of hydrogen that of the charged specimen, 127*b*^3^, is only a little smaller than the uncharged specimen, 133 *b*^3^. This is fully consistent with our calculations which show that ${\bar{v}}_{{\rm{dis}}}$ decreases to approach that of pure *α*-Fe at the “30 appm anomaly”. At this stage of the work we cannot unequivocally associate our “30 appm anomaly” with the maximum in the activation volume found by Wang *et al*.^10^ and plotted in Fig. 7. This is firstly because we have not made experiments over a range of hydrogen concentrations and secondly because of the difficulty in establishing a quantitative measure of *C*_H_ in both the specimens measured by Wang *et al*.^10^ and in our simulations. The problem is the same in either case—we are using the amount of hydrogen trapped at defects as a surrogate for the background hydrogen concentration, *C*_H_. In Wang *et al*.’s measurements *C*_H_ is inferred from the cathodic current density using a number of assumptions on the hydrogen diffusivity and the distribution and depths of traps. It is clear from their method that an uncertainty of about 30 meV in trap depth leads to a factor of 13 as the ratio of the upper and lower estimates of *C*_H_. Therefore while they find the anomaly to be at 18 appm, by their own methodology and using reasonable uncertainties in trap depth this may have been as large as 200 appm. The same applies to our assertion that the anomaly occurs at 30 appm. We use the whole range of trap depths in the McLean isotherm to infer the nominal hydrogen concentration that corresponds to a particular simulation. For example the depth of the E_2_/E_3_ basin is calculated in DFT to be 256 ± 32 meV^9^. Given this uncertainty, our assignment of 30 appm of the anomaly is our best estimate of a value that may range between 12 and 105 appm. In spite of this we assert that the “30 appm anomaly” is real (although perhaps not well-named) and will have great significance in both the interpretation of experiments and in the establishment of non trivial models for dislocation velocity to be used in multiscale models of hydrogen embrittlement. A key finding here is that the *macroscopic* measurements that indicate the anomaly can be traced *microscopically* to the screw dislocation core transformation brought about by hydrogen, Fig. 2.

### Implication for dislocation cell formation

**Figure 13:**
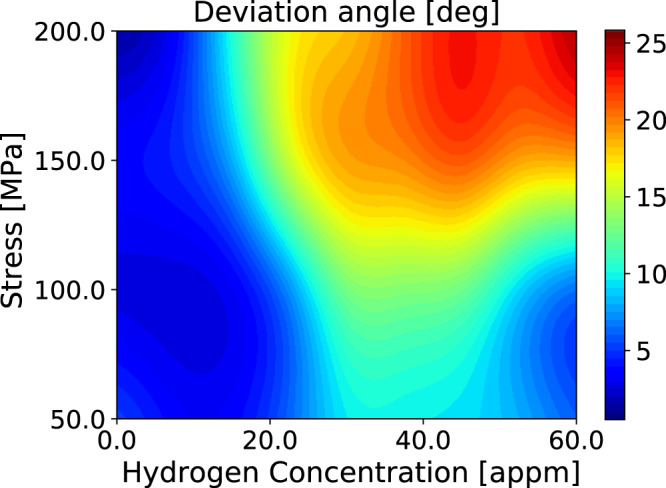
The angle describing the deviation of a long straight $\frac{1}{2}[111]$ screw dislocation from the primary glide plane, after moving over a distance of 50 nm, as a function of *C*_H_ and resolved shear stress.

Our transmission electron microscopy observations of dislocation structures of both hydrogen charged and uncharged specimens in *α*-Fe show that the homogeneous dislocation forest existing in hydrogen free samples transforms into cell walls that separate relatively dislocation free regions. The cell walls can be regarded as dense dislocation tangles. The driving force for cell wall formation arises from the reduction in the total elastic energy of the dislocations due to their clustering. TEM images have shown that the volume of the dislocation free zones and the density of the tangled structures increase with increasing *C*_H_ in the interval 0–25 appm^10^. The physics that lies behind dislocation reorganisation due to hydrogen is not yet well understood. It has been commonly accepted that a requirement for cell formation is that dislocations have sufficient mobility out of their slip plane^29^. Therefore, whether cells form or not depends on factors which determine the ease with which dislocations cross slip or climb. The present SCkMC simulations and experiments show that the probability for formation of kink pairs in secondary slip planes and dislocation segments which glide out of the primary slip plane increases with *C*_H_. The angle describing the deviation of the dislocation from the primary slip plane as a function of applied stress and *C*_H_ is shown in Fig. 13. Again, at moderate stresses where glide is dominated by kink pair formation enthalpy, we see an anomaly near *C*_H_ = 30 appm near the EC–HC core transformation, where *E*_kp_ is small and kink pair activation is prolific on the cross slip planes. SCkMC simulations show that the dislocation mobility out of the primary slip plane increases significantly for *C*_H_ > 10 appm and applied stresses higher than 100 MPa. This result of the SCkMC simulations agrees with TEM observations indicating an increase of the density of the tangled structures with increase of hydrogen concentration.

The appearance of dislocation cell structures and the role of hydrogen is not yet firmly established in the experimental literature. Our own point of view is that we expect that both in uncharged and hydrogen-charged *α*-Fe dislocation cell formation is to be expected in some circumstances. We expect (*i*) that the *scale* of the cell structure is expected to be smaller as *C*_H_ is increased; and (*ii*) that the critical dislocation density at which the transition occurs from uniform distribution to a cell structure decreases as *C*_H_ is increased. Most experimental evidence on the role of hydrogen on cell formation comes from fatigue testing^30–33^. In most of these studies cell formation is found in both uncharged and hydrogen-charged specimens, while there is some evidence that supports our theory that hydrogen serves to decrease the size-scale of the cell structure^30^. On the other hand, the state of stress and the extent of plasticity are very different under fatigue compared to uniaxial stress relaxation. In addition, our viewpoint is supported by dislocation cell formation observed in stress relaxation testing of uncharged and hydrogen-charged *α*-Fe^10,34^.

## Conclusions

1. We demonstrate a new self consistent kinetic Monte Carlo scheme that is able to calculate average dislocation velocity of long straight $\frac{1}{2}[111]$ screw dislocations in pure and hydrogen loaded *α*-Fe. The self consistency arises because of the parametric dependences of the speed on the kink pair formation enthalpy, and the kink pair formation enthalpy on the speed.
2. As predicted in previous work^14^, we find that an effect of hydrogen is to generate large quantities of debris behind a moving screw dislocation, even at room temperature. This is a consequence of kink collisions on different slip planes.
3. The predicted debris has now been found in TEM images of hydrogen loaded *α*-Fe, following tensile stress relaxation testing.
4. We have identified what we call the “30 appm anomaly”. This corresponds to the hydrogen concentration at which there is a core transformation of the screw dislocation from easy core to hard core configuration. At the critical concentration, the Peierls barrier and kink pair formation enthalpy approach close to zero, before increasing as *C*_H_ increases beyond 30 appm and the barrier for glide subsequently appears at the EC state. Signatures of the anomaly are, (*i*) very frequent kink pair production and creation of self pinning jogs, (*ii*) a *minimum* in ${\bar{v}}_{{\rm{dis}}}$ due to the profilific creation of debris (see Figs. 5, 6) (*iii*) a plateau, or minimum in the effective activation volume for slip (Fig. 7).

## Further work

We have identified what we call the “30 appm anomaly” and made contact with published stress relaxation experiments. Our principal conclusion is that this *macroscopic* effect is real and is associated with the *microscopic* phenomenon of a hydrogen-induced screw dislocation core transformation. We have shown that a precise determination of the nominal hydrogen concentration at which the anomaly will occur is problematic both in theory and in experiment. Future work is needed to make a systematic series of stress relaxation experiments in order to make the definitive association of the anomaly with the non monotonic change of activation volumes with hydrogen concentration; and these experiments are planned. It is also a matter of further work to investigate the role of hydrogen and other interstitials such as carbon and nitrogen on the dynamics of cell formation and the development of cellular and sub-grain microstructures in simple uniaxial loading, and to make the connections to observed microstructures under hydrogen-induced fatigue failure.
